# Supplementary material for: Flexible Perfluoropolyethers-Functionalized CNTs-Based UHMWPE Composites: A Study on Hydrogen Evolution, Conductivity and Thermal Stability
Source: Materials (Basel). 2022 Oct 3;15(19):6883. doi: 10.3390/ma15196883 (PMC9571002; doi:10.3390/ma15196883)
Supplement: Supplementary file 1 [file materials-15-06883-s001.zip › materials-1843308-supplementary.pdf]

materials

MDPI

Supplementary Materials

Flexible Perfluoropolyethers-Functionalized CNTs-Based UHMWPE Composites: A Study on Hydrogen Evolution, Conductivity and Thermal Stability

Maurizio Sansotera [1,2,*], Valeria Marona [1,2], Piergiorgio Marziani [1], Nadka Tzankova Dintcheva [3,4], Elisabetta Morici [3,5], Rossella Arrigo [3,4], Gianlorenzo Bussetti [6], Walter Navarrini [1,2] and Luca Magagnin [1,2]

Table S1. Electrical resistivity values of CNTs-based nanocomposites.

	Resistivity [Ω m]					
	17.2 kPa			32.5 kPa		
Nano-filler [wt.%]	UHMWPE/ CNTs	UHMWPE/ CNTs-PFPE50	UHMWPE /CNTs-PFPE100	UHMWPE/ CNTs	UHMWPE/ CNTs-PFPE50	UHMWPE/ CNTs-PFPE100
0.5	$3.73 \cdot 10^{8}$ $\pm 1.40 \cdot 10^{8}$	$9.31 \cdot 10^{6}$ $\pm 1.61 \cdot 10^{6}$	$2.81 \cdot 10^{6}$ $\pm 1.49 \cdot 10^{6}$	$1.60 \cdot 10^{8}$ $\pm 6.38 \cdot 10^{7}$	$7.20 \cdot 10^{5}$ $\pm 1.77 \cdot 10^{5}$	$1.12 \cdot 10^{6}$ $\pm 4.63 \cdot 10^{5}$
1	$4.34 \cdot 10^{8}$ $\pm 5.55 \cdot 10^{7}$	$1.95 \cdot 10^{4}$ $\pm 4.06 \cdot 10^{3}$	$4.38 \cdot 10^{3}$ $\pm 4.20 \cdot 10^{2}$	$2.75 \cdot 10^{8}$ $\pm 1.29 \cdot 10^{7}$	$4.30 \cdot 10^{3}$ $\pm 4.97 \cdot 10^{2}$	$3.89 \cdot 10^{3}$ $\pm 1.27 \cdot 10^{2}$
3	$1.44 \cdot 10^{5}$ $\pm 2.12 \cdot 10^{4}$	$3.59 \cdot 10^{2}$ $\pm 2.24 \cdot 10^{1}$	$2.06 \cdot 10^{2}$ $\pm 4.72 \cdot 10^{2}$	$1.18 \cdot 10^{5}$ $\pm 1.41 \cdot 10^{4}$	$1.35 \cdot 10^{2}$ $\pm 1.85 \cdot 10^{1}$	$1.37 \cdot 10^{2}$ $\pm 2.75 \cdot 10^{1}$
5	$1.02 \cdot 10^{3}$ $\pm 2.30 \cdot 10^{2}$	$2.89 \cdot 10^{2}$ $\pm 1.03 \cdot 10^{2}$	$4.16 \cdot 10^{2}$ $\pm 1.97 \cdot 10^{2}$	$1.03 \cdot 10^{3}$ $\pm 2.27 \cdot 10^{2}$	$1.06 \cdot 10^{2}$ $\pm 1.10 \cdot 10^{1}$	$1.73 \cdot 10^{2}$ $\pm 6.16 \cdot 10^{1}$
0	$8.98 \cdot 10^{7} \pm 1.14 \cdot 10^{8}$			$1.325 \cdot 10^{8} \pm 1.204 \cdot 10^{8}$		

Table S2. Electrical resistivity values of CB-based composites.

	Resistivity [Ω m]			
	17.2 kPa		32.5 kPa	
Nano-filler [wt.%]	UHMWPE/ CB	UHMWPE/ CB-PFPE	UHMWPE/ CB	UHMWPE/ CB-PFPE
0.5	$1.97 \cdot 10^{8}$ $\pm 4.31 \cdot 10^{7}$	$1.85 \cdot 10^{8}$ $\pm 2.12 \cdot 10^{8}$	$2.17 \cdot 10^{8}$ $\pm 3.87 \cdot 10^{7}$	$2.65 \cdot 10^{8}$ $\pm 4.14 \cdot 10^{7}$
1	$2.38 \cdot 10^{4}$ $\pm 5.52 \cdot 10^{3}$	$5.23 \cdot 10^{5}$ $\pm 9.21 \cdot 10^{4}$	$9.52 \cdot 10^{3}$ $\pm 8.64 \cdot 10^{2}$	$2.99 \cdot 10^{5}$ $\pm 5.45 \cdot 10^{4}$
3	$1.37 \cdot 10^{3}$ $\pm 3.94 \cdot 10^{2}$	$1.53 \cdot 10^{3}$ $\pm 1.79 \cdot 10^{2}$	$6.17 \cdot 10^{2}$ $\pm 1.04 \cdot 10^{2}$	$9.01 \cdot 10^{2}$ $\pm 8.93 \cdot 10^{1}$

5	$4.77 \cdot 10^{2}$ $\pm 3.98 \cdot 10^{1}$	$1.09 \cdot 10^{3}$ $\pm 2.58 \cdot 10^{2}$	$2.61 \cdot 10^{2}$ $\pm 4.12 \cdot 10^{1}$	$3.93 \cdot 10^{2}$ $\pm 8.36 \cdot 10^{1}$
0	$8.98 \cdot 10^{7} \pm 1.14 \cdot 10^{8}$		$1.325 \cdot 10^{8} \pm 1.20 \cdot 10^{8}$	
